# LALAPG variant of the Human Contraception Antibody (HCA) reduces Fc-mediated effector functions while maintaining sperm agglutination activity

**DOI:** 10.1371/journal.pone.0282147

**Published:** 2023-03-30

**Authors:** Emilie Mausser, Ellena Nador, Joseph A. Politch, Michael R. Pauly, Jai G. Marathe, Thomas R. Moench, Larry Zeitlin, Kevin J. Whaley, Deborah J. Anderson

**Affiliations:** 1 Division of Medical Sciences, Boston University Aram V. Chobanian & Edward Avedisian School of Medicine, Boston, Massachusetts, United States of America; 2 Department of Medicine, Boston University Aram V. Chobanian & Edward Avedisian School of Medicine, Boston, Massachusetts, United States of America; 3 ZabBio, Inc., San Diego, California, United States of America; 4 Mapp Biopharmaceutical, Inc., San Diego, California, United States of America; 5 Mucommune, Chapel Hill, North Carolina, United States of America; Nelson Mandela African Institute of Science and Technology, UNITED REPUBLIC OF TANZANIA

## Abstract

High rates of unintended pregnancies worldwide indicate a need for more accessible and acceptable methods of contraception. We have developed a monoclonal antibody, the Human Contraception Antibody (HCA), for use by women in vaginal films and rings for contraception. The divalent F(ab’)2 region of HCA binds to an abundant male reproductive tract-specific antigen, CD52g, and potently agglutinates sperm. Certain other antibody activities mediated by the Fc region such as mucus trapping, complement-dependent cytotoxicity (CDC) and antibody-dependent cellular phagocytosis (ADCP) could have beneficial or negative effects. The purpose of this study was to document HCA Fc effector functions and determine whether an engineered variant of HCA with a modified Fc region, HCA-LALAPG, retains desirable contraceptive activity while minimizing Fc-mediated effects. Fab and Fc functions were compared between HCA and HCA-LALAPG. Fab activity was assessed using sperm agglutination and modified swim-up ("sperm escape”) assays. Fc functions were assessed by CDC (sperm immobilization), ADCP, and cervical mucus penetration assays. HCA and HCA-LALAPG showed equivalent activity in assays of Fab function. In the assays of Fc function, HCA supported strong CDC, ADCP, and sperm trapping in cervical mucus whereas HCA-LALAPG demonstrated little to no activity. HCA and the HCA-LALAPG variant were both highly effective in the sperm agglutination assays but differed in Fc mediated functions. Use of the HCA-LALAPG variant for contraception in women could reduce antibody-mediated inflammation and antigen presentation but may have reduced contraceptive efficacy due to much weaker sperm trapping in mucus and complement-dependent sperm immobilization activity.

## Introduction

The United Nations reports that almost half of all pregnancies are unintended due to lack of or incorrect use of effective contraception [1]. This points to the need for better contraception education and services, and access to more acceptable, easy to use contraception methods. Male contraception and new non-hormonal products for women may fill this gap and allow greater control of reproduction. A new contraceptive product that is low-cost, easily accessible, user-controlled, non-invasive, reversible, safe, and unobtrusive during intercourse might be popular among women who presently do not consistently use effective contraception methods due to their high cost, limited access and undesirable side effects [2, 3]. A promising new contraceptive candidate is the Human Contraception Antibody (HCA), an antisperm antibody currently in clinical development as a topical non-hormonal contraceptive for women [4]. HCA binds to a glycoprotein, CD52g, on the surface of sperm, and has been shown to rapidly agglutinate sperm and prevent their progression through the female reproductive tract.

Over 100 antibody-based drugs have been approved by the US Food and Drug Administration (FDA) with many more currently in clinical trials [5]. While a majority of these are for treatment of cancer and autoimmune disorders, there is growing interest in using antibodies to prevent and treat a wider range of indications, including infectious diseases and unintended pregnancy. We are currently using a transgenic *Nicotiana*-based production platform to produce monoclonal antibodies (mAbs) for reproductive health. Our group recently tested a multipurpose prevention technology (MPT) product, a vaginal film containing *Nicotiana*-based mAbs against human immunodeficiency virus 1 (HIV-1) and herpes simplex virus type 2 (HSV-2), in a Phase 1 clinical trial. The film, MB66, was safe and maintained antiviral activity in the vaginal mucosa for up to 24 hours [6]. HCA was recently formulated into a vaginal film and tested in a Phase 1 clinical trial where it demonstrated safety and contraceptive efficacy in postcoital tests (Clinical Trial Registration: NCT04731818). The HCA vaginal films used in the Phase I clinical trials were manufactured at 20 mg per film. The median concentration of HCA in the FRT 4 hours after film application was 500 μg/mL (range 20–2,400 μg/mL).

The majority of mAbs produced for clinical applications, including our lead HIV-1, HSV-2, and HCA mAbs, belong to the IgG1 antibody subclass [7]. IgG1 mAbs bind to target antigens through the variable (Fab) region and can participate in other effector functions through the constant (Fc) region. Many immune cells express receptors that specifically bind to the Fc region of IgGs (FcγRs). FcγRs recognize IgG-coated targets such as opsonized pathogens or immune complexes; cross-linking leads to internalization and activation of downstream signaling cascades. Two effector cell activities elicited by FcγR activation include antibody-dependent cellular phagocytosis (ADCP) and antibody-dependent cellular cytotoxicity (ADCC). The IgG1 Fc region also contains binding sites for the complement component C1q which can initiate the complement cascade leading to complement-dependent cytotoxicity (CDC) [8]. IgG Fc can also bind to the neonatal Fc receptor, FcRn, that regulates antibody recycling [9]. The Fc region may also play a role in trapping pathogens and sperm in mucus. Jager et al. were the first to report that sperm coated with antisperm antibodies become trapped in cervical mucus and that the Fc region may play a role [10]. A number of recent studies provide further evidence that multiple weak bonds between IgG-Fc and mucins trap IgG-coated pathogens and sperm in mucus [11].

**Fig 1:**
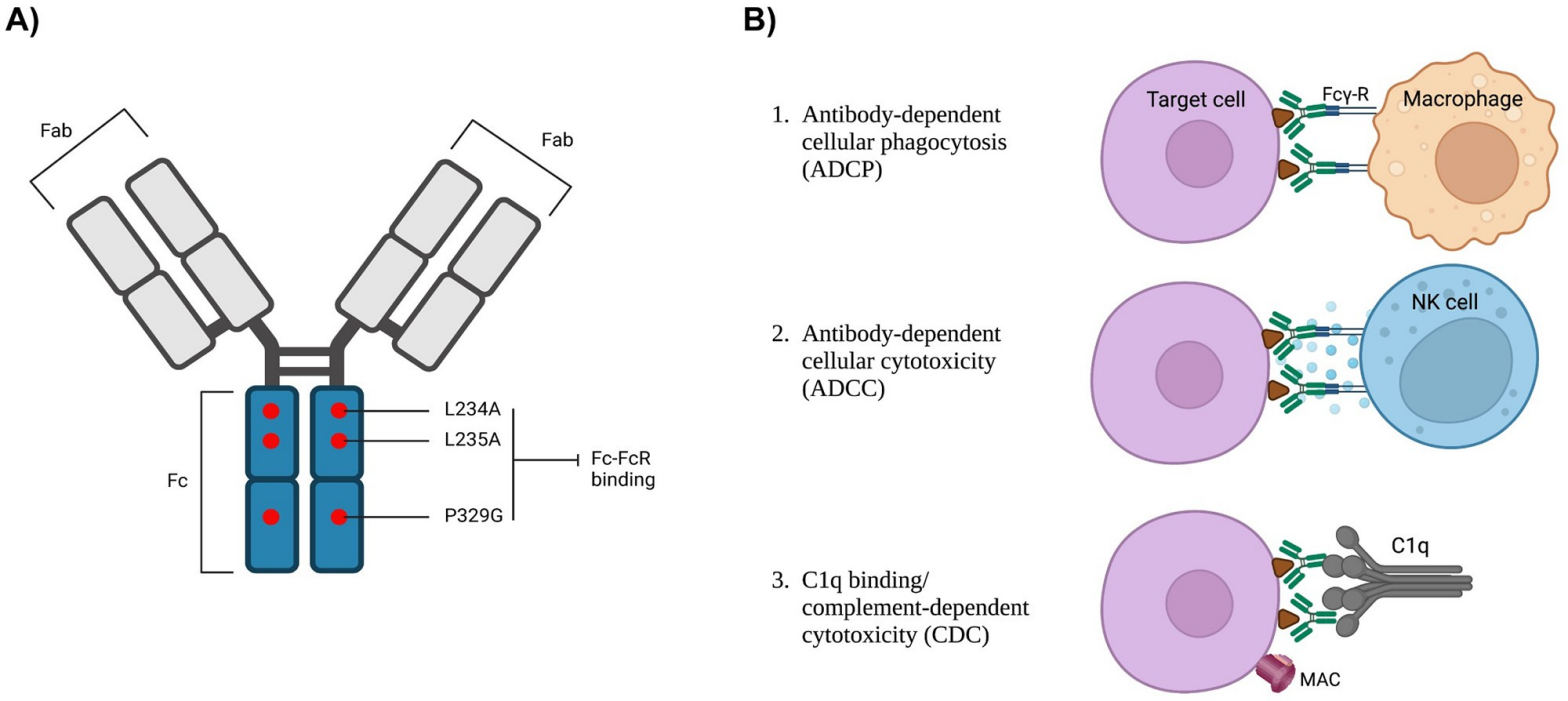
LALA-PG mutation and Fc functions of IgG. A) Diagram of Fc-engineered HCA variant. Red dots on the Fc region indicate approximate locations of the three amino acid mutations and their respective residue substitutions. B) Fc functions inhibited by the Fc LALA-PG mutation. Created with BioRender.com.

Fc-mediated antibody functions are desirable for certain applications such as mAb-mediated pathogen clearance, but they may be undesirable in other situations. ADCC and CDC can elicit inflammation [12], and ADCP may stimulate downstream adaptive immune responses by facilitating antigen presentation [13]. In the case of HCA vaginal film, an Fc-mediated inflammatory response could enhance HIV transmission [14], and presentation of sperm antigens by immune cells in the female reproductive tract following ADCP could potentially lead to antisperm immunity and infertility [15]. Fc interactions and consequent effector cell functions can be tailored through Fc engineering [16], and a number of genetically engineered Fc variants have been introduced. One such variant, LS, contains a mutation associated with improved binding to the FcRn receptor and increased antibody serum half-life which would require less frequent dosing of an antibody-based drug [17]. Fc engineering can also be used to reduce effector function to minimize unwanted inflammation and acquired immunity induced by antibody-based drugs. The most common of these Fc designs, known as LALAPG, includes three point mutations: L234A, L235A, and P329G [18] (Fig 1). LALAPG variants inhibit binding to FcγRs and C1q while FcRn binding and Fc stability remain unaffected [19]. Effects of LALAPG mutations on Fc-mucus interactions have not been described. Several antibodies with LALAPG mutations are currently undergoing testing in clinical trials [20]. Engineering the Fc region of antibody-based drugs can help tailor the drug for a specific desired effect.

As part of clinical development, various engineered versions of HCA are being tested to determine whether they enhance antibody potency and reduce potential side effects. HCA variants have been engineered with increased valency to enhance agglutination potency [21]. A LALAPG HCA variant has also been developed and is being explored as a means to reduce the potential for undesirable Fc-mediated side effects such as inflammation and acquired immunity [22, 23]. In this study, we compared Fab and Fc functions of HCA and HCA-LALAPG to more fully elucidate the mechanisms of action of HCA, and to inform the optimal design of this antisperm antibody for contraceptive use.

## Materials and methods

### 1. Antibodies

#### Human Contraception Antibody (HCA)/HCA-LALAPG

HCA, a human anti-CD52g mAb, was produced in *Nicotiana benthamiana* from the variable region sequence of H6-3C4 [24] and an optimized human IgG1 constant region as previously described [4]. This IgG1 form of HCA is also referred to as HC4-N. Transgenic *Nicotiana* plants with knockdown of fucosyl- and xylosyl-transferases allowed for the insertion of humanized glycans as previously described [25]. HCA-LALAPG was produced in the *Nicotiana* mAb platform from a DNA template with three amino acid substitutions in the Fc region: L234A, L235A, and P329G.

#### Campath-1

Campath-1, or alemtuzumab, is a humanized IgG monoclonal antibody used for cancer immunotherapy (ThermoFisher Scientific Cat# MA5-16999, Waltham, MA, USA, RRID: AB_2538471). Campath-1 binds to a peptide epitope present on CD52, an antigen found on normal and malignant hematopoietic cells, and CD52g, a heavily glycosylated male reproductive tract-specific form of CD52. HCA binds to a carbohydrate epitope only found on CD52g. Campath-1 was used as a positive control for the sperm phagocytosis assay as it has been shown to bind to sperm [4] and activate ADCP by macrophages [26].

#### VRC01

VRC01-N, a broadly neutralizing anti-HIV-1 IgG1 antibody [27], was also produced in *Nicotiana*. It was used as an isotype control in the sperm phagocytosis assay.

### 2. Sample collection and processing

This study was approved by the Institutional Review Board at Boston University Medical Campus (Human Subjects Protocols H36843 and H41454). All participants provided written informed consent prior to participation.

#### Semen

Human semen samples were obtained from healthy men aged 18–45 after at least two days of sexual abstinence. All samples met the WHO criteria for fertility [28]. Samples were processed within one hour of collection and liquefaction at 32°C. All sperm incubations were conducted at 32°C as they retain maximum viability at this temperature. This is approximately the temperature within the testes, which is lower than normal body temperature. Whole semen was overlaid on an equal volume of 90% ISolate density gradient (FUJIFILM Irvine Scientific; Santa Ana, CA, USA) and centrifuged at 300 x g for 20 minutes. The sperm pellet containing progressively motile sperm was re-suspended in Multipurpose Handling Medium (MHM; FUJIFILM Irvine Scientific; Santa Ana, CA, USA). Sperm concentration and motility parameters were assessed using a Computer-Assisted Sperm Analysis system (CASA; Human Motility II software, CEROS II, Hamilton Thorne, Beverly, MA, USA). Sperm concentrations were adjusted to 25–40 x10^6^/mL for use in the assays.

#### Cervical mucus

Midcycle cervical mucus was collected by a trained physician from reproductive-aged women within 48 hours of a positive ovulation test (Digital Ovulation Predictor Kit, Clearblue). An endocervical pipelle (Aspirette Endocervical Pipelle, Cooper Surgical, Trumbull, CT, USA) was used to aspirate pure cervical mucus directly from the endocervical canal. None of the women were on hormonal contraception. Ovulation was confirmed for all women by evaluation of the mucus (clear with spinnbarkeit characteristics), and by estrogen and progesterone levels in blood. Cervical mucus was diluted 1:3 in PBS and aspirated into flat capillary tubes (Borosilicate Capillary Glass Slide, 0.30x3.0 mm, 50 mm, Electron Microscopy Sciences, Hatfield, PA, USA). Both ends of the tube were sealed with parafilm, and samples were stored at 4°C for up to 5 days. Cervical mucus samples from four different donors were used for this study.

### 3. Agglutination kinetics assay

The effects of HCA and HCA-LALAPG on sperm agglutination were determined using the sperm agglutination kinetic assay as described previously [4]. A 2 μL sample of sperm cell suspension in MHM was added to a Multi-Spot microscope slide (ThermoScientific, Waltham, MA, USA). An equal volume of antibody diluted in MHM was mixed into the well containing sperm. Agglutination of sperm was observed in real time on an Olympus inverted microscope at 10x. The time elapsed to achieve complete agglutination (i.e., 100% agglutinated sperm), was recorded. Since it has been reported that sperm can reach the endocervical canal in 3–5 minutes, a cutoff time of 2.5 min was used for the assay [4, 29, 30]. Data points were acquired in triplicate and the experiment was repeated three times using semen from different donors.

### 4. Sperm escape assay

The percentage of progressively-motile sperm that escaped sperm agglutinates after antibody exposure was quantified as described previously [4]. Sperm were added to PCR tubes containing HCA, HCA-LALAPG or medium alone (control) and incubated at room temperature at a 45-degree angle for 5 minutes. A 2.5 μL sample was taken from the top millimeter of the tube and placed in a 4-chamber slide (Microcell 15424, Vitrolife, San Diego, CA, USA) for counting on the CASA. Data points were acquired in triplicate and the experiment was repeated using semen samples from three different donors. Data were presented as percent of control (medium alone).

### 5. Complement-dependent sperm immobilization test

The sperm immobilization test was performed as described previously [4, 31, 32]. Freshly collected serum, used as a complement source, was stored at -80°C and thawed on ice before initiating the assay. Briefly, 2.5 μL of washed sperm were sensitized with 5 μL of human serum for 5 minutes, then 25 μL of diluted antibody was added and the samples were incubated in 4-chambered slides inside a wet box for 60 minutes at 32°C. Resulting sperm motility was measured using the CASA. Heat-inactivated complement (HiC), used as a negative control, was prepared by heating serum at 56°C for 30min. Experiments were performed three times with samples from different semen donors.

### 6. Cervical mucus penetration assay

Capillary tubes containing cervical mucus were warmed to 32°C and each 1 cm interval on the capillary tube was marked. Motile sperm were prepared as described above but re-suspended at a concentration of 25–40 x10^6^/mL in seminal plasma instead of MHM. Antibodies were diluted to 12.5 μg/mL in MHM. Ten μL of diluted antibody or MHM alone (control) were added to one end of the capillary tube, and the opposite end was then sealed with parafilm. The open end of the capillary tube was inserted horizontally into a tube containing 100 μL of sperm so that the sperm would swim through the antibody or medium (control) before entering the column of cervical mucus. Tubes were incubated in a humidified chamber at 32°C. At 30-, 60- and 90-minute time points, sperm were assessed at 1, 2, 3 and 4 cm depths using the CASA. Progressive sperm in each section were scored by manual review of the CASA output by a trained researcher.

### 7. Antibody-dependent sperm phagocytosis assay

U937 (CRL-1593.2, obtained directly from ATCC) pro-monocytes were seeded onto sterile glass coverslips in 6-well plates at a density of 0.5 x 10^6^ cells/mL in 3 mL of RPMI 1640 complete medium (Gibco, ThermoScientific, Waltham, MA, USA; supplemented with +L-glutamine, 10%FBS, 1% Penicillin-Streptomycin). Cells were treated with 100 ng/mL phorbol-12-myristate-13-acetate (PMA) to induce differentiation into macrophages [33] and were incubated under an atmosphere of 5% CO_2_ at 37°C for 48–72 hours, after which they became adherent to the coverslips.

The sperm phagocytosis assay was performed as described by Oren-Benaroya et al. with modifications [34]. In brief, the growth medium in the macrophage cultures was discarded and 1 X 10^6^ sperm in either MHM or in MHM supplemented with 50 μg/mL antibody were added to each well. Following a 30-minute incubation at 37°C and 5% CO_2_, the macrophage cultures were washed 3X with PBS. After another 30-minute incubation at 37°C in PBS, the macrophage cultures were incubated in trypsin for 5 minutes to remove non-specifically bound spermatozoa. The coverslips were treated with Differential Quik III fixative solution (Polysciences, Warrington, PA, USA), stained with Differential Quik III stain solution I and II, and washed in tap water before being mounted to glass slides. Macrophages were observed under a light microscope at X200 magnification, and the number of spermatozoa associated with 100–300 macrophages was counted for each treatment group. Number of internalized sperm per macrophage was also assessed and characterized as sperm partially engulfed, either head or tail, or wholly engulfed by a macrophage. The experiment was repeated three times using semen samples from different donors.

### 8. Statistical analyses

Data were analyzed by either repeated measures analysis of variance (ANOVA) or mixed-effects analysis. A significant analysis was followed by either post hoc Tukey or Sidak multiple comparison tests as indicated by the type of analysis. If continuous variables were not normally distributed, they were log (natural) transformed prior to analysis. The equality of variances of the pairwise differences of within subject conditions was assessed through a test of sphericity (Geisser-Greenhouse’s epsilon). GraphPad Prism (Version 9.4.1; GraphPad Software Inc.; San Diego, CA, USA) and JMP Pro (Version 15.2.0; SAS Institute Inc., Cary, NC, USA) were used for statistical analysis and graph creation. Differences were considered to be statistically significant when p<0.05.

## Results

### Both HCA and HCA-LALAPG robustly agglutinate sperm

**Fig 2:**
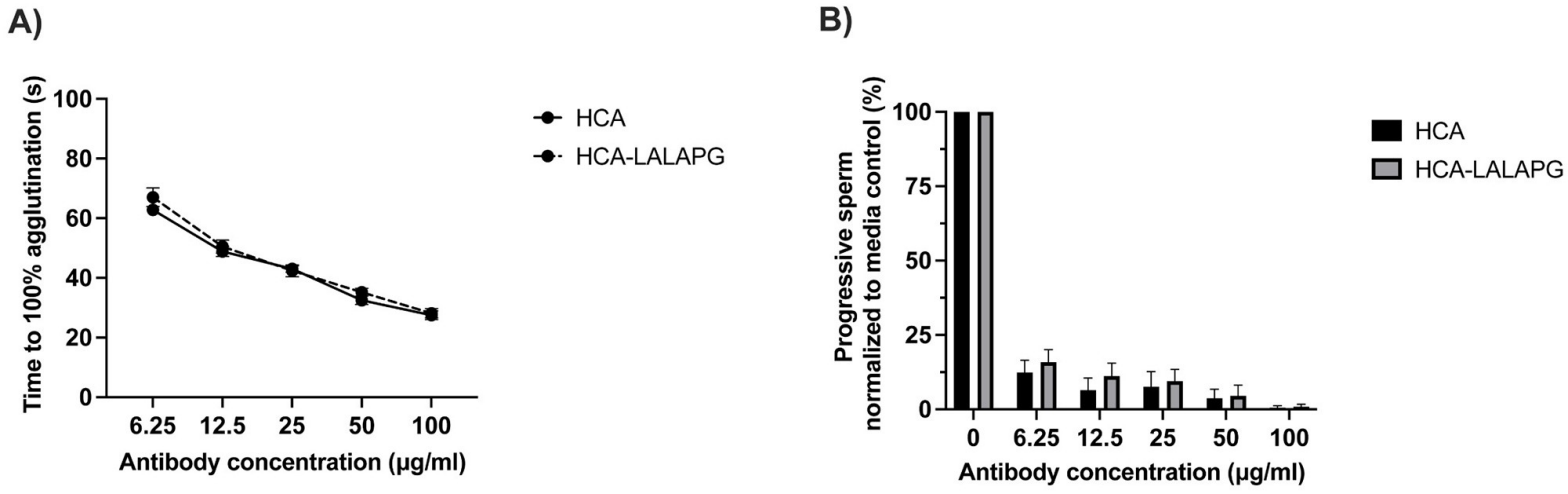
Sperm agglutination by HCA and HCA-LALAPG. A) Agglutination kinetics assay shows similar dose response curves for HCA and HCA-LALAPG. B) Sperm escape assay shows similar percent of progressive sperm relative to the medium-only control for HCA and HCA-LALAPG at all observed antibody concentrations. Data are expressed as means ± SEM of three independently performed experiments. Statistical analyses were conducted using repeated measures two-way ANOVA of log-transformed data followed by Sidak multiple comparisons test. No significant differences were found between the two groups for either assay (p > 0.6). SEM, standard error of the mean.

Because agglutination of sperm relies on the Fab portion of HCA, Fc mutations would not be expected to impact agglutination potency. We compared the agglutination function of parent HCA and HCA-LALAPG using agglutination kinetics and sperm escape assays. As expected, the two antisperm antibodies did not differ in time to 100% agglutination over a wide concentration range (p > 0.6, Fig 2A). In fact, the two concentration curves appeared to be superimposed. At 100 μg/mL, both antibodies agglutinated 100% of sperm within 30 seconds. At the lowest concentration, 6.25 μg/mL, the antibodies agglutinated 100% of sperm within 60 seconds, well below the 150 second time cutoff. Likewise, HCA and HCA-LALAPG did not differ significantly in percent of progressively motile sperm cells in the sperm escape assay (sperm that “escaped” agglutination), following a 5-minute incubation of sperm and antibody (p > 0.7, Fig 2B). At an antibody concentration of 100 μg/mL, the number of progressively motile sperm for both the HCA and HCA-LALAPG antibodies was <1% of the medium-only control; the percent increased to 12–15% at an antibody concentration of 6.25 μg/mL.

### HCA immobilizes sperm in the presence of complement whereas HCA-LALAPG does not

**Fig 3:**
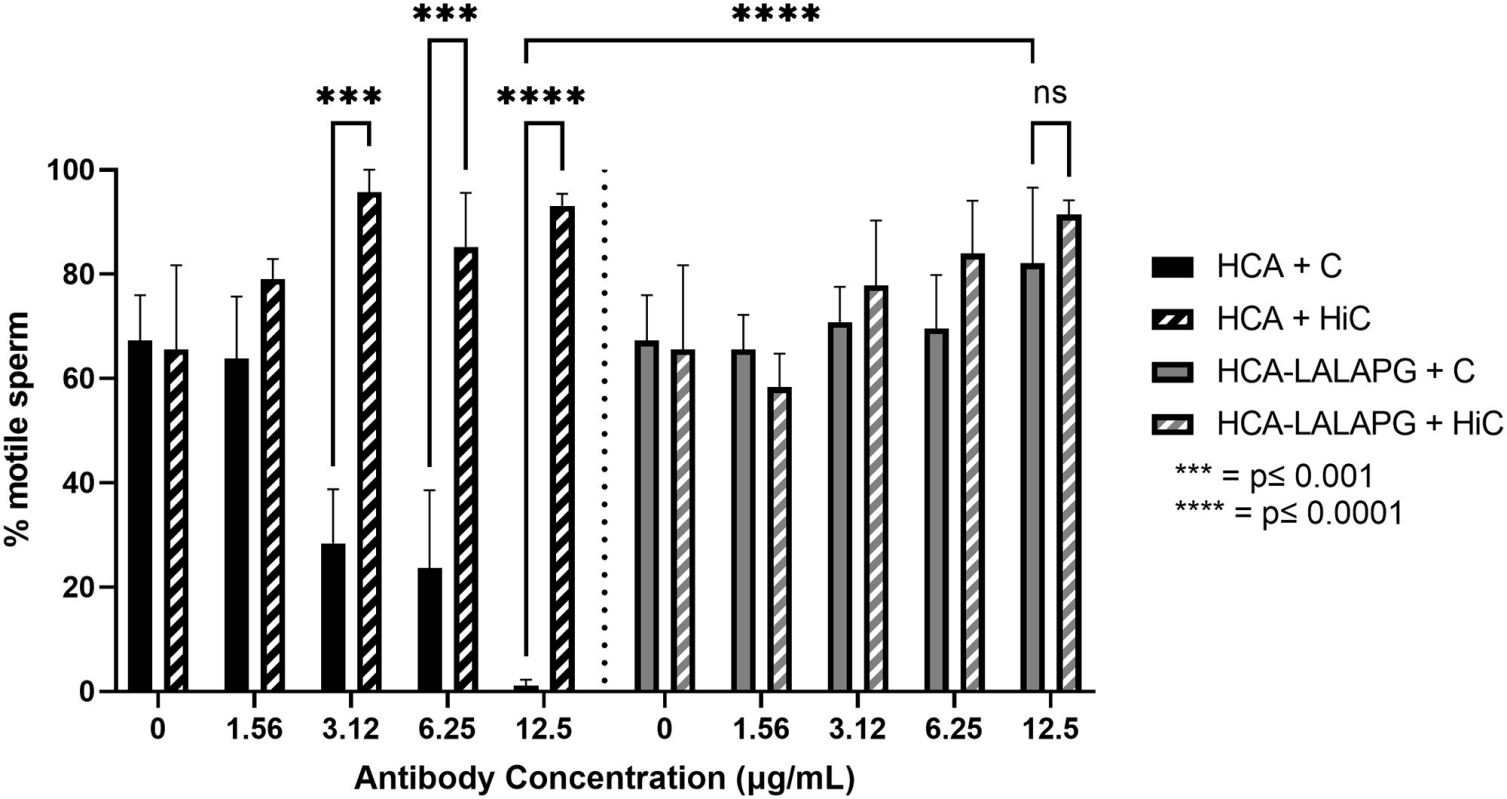
Sperm immobilization test with HCA and HCA-LALAPG. HCA significantly immobilized sperm in the presence of complement (C) in a concentration-dependent manner (p = <0.0001, 0.0005, 0.0001 for 12.5, 6.25, and 3.12 μg/mL, respectively). Heat-inactivated complement (HiC) was used as a negative control and did not cause immobilization. HCA-LALAPG did not significantly affect sperm motility with either C or HiC (p = 0.9105). Data are shown as mean ± SEM and is representative of three independently performed experiments. Statistical analyses were conducted using a two-way ANOVA followed by a Tukey multiple comparisons test. Results were significant when p ≤ 0.05. (*** = p ≤ 0.001, **** = p ≤ 0.0001).

HCA significantly immobilized sperm in the presence of complement; sperm motility was significantly reduced at HCA concentrations ranging from 12.5 to 3.12 μg/mL (Fig 3, p <0.001). At these concentrations, HCA agglutinated sperm but there were sufficient numbers of non-agglutinated sperm to enable motility assessment. Agglutinated sperm were also immobilized by antibody and complement. Without complement, agglutinated sperm retain movement though they do not show forward progression (“wiggling in place”). In complement-mediated immobilization, the sperm in agglutinates are static and show no movement. Sperm motility was not affected by HCA in the presence of HiC. HCA-LALAPG, which is unable to bind the C1q complement protein, did not immobilize sperm at any antibody concentration tested (p = 0.9105).

### HCA traps sperm in cervical mucus whereas HCA-LALAPG does not

**Fig 4:**
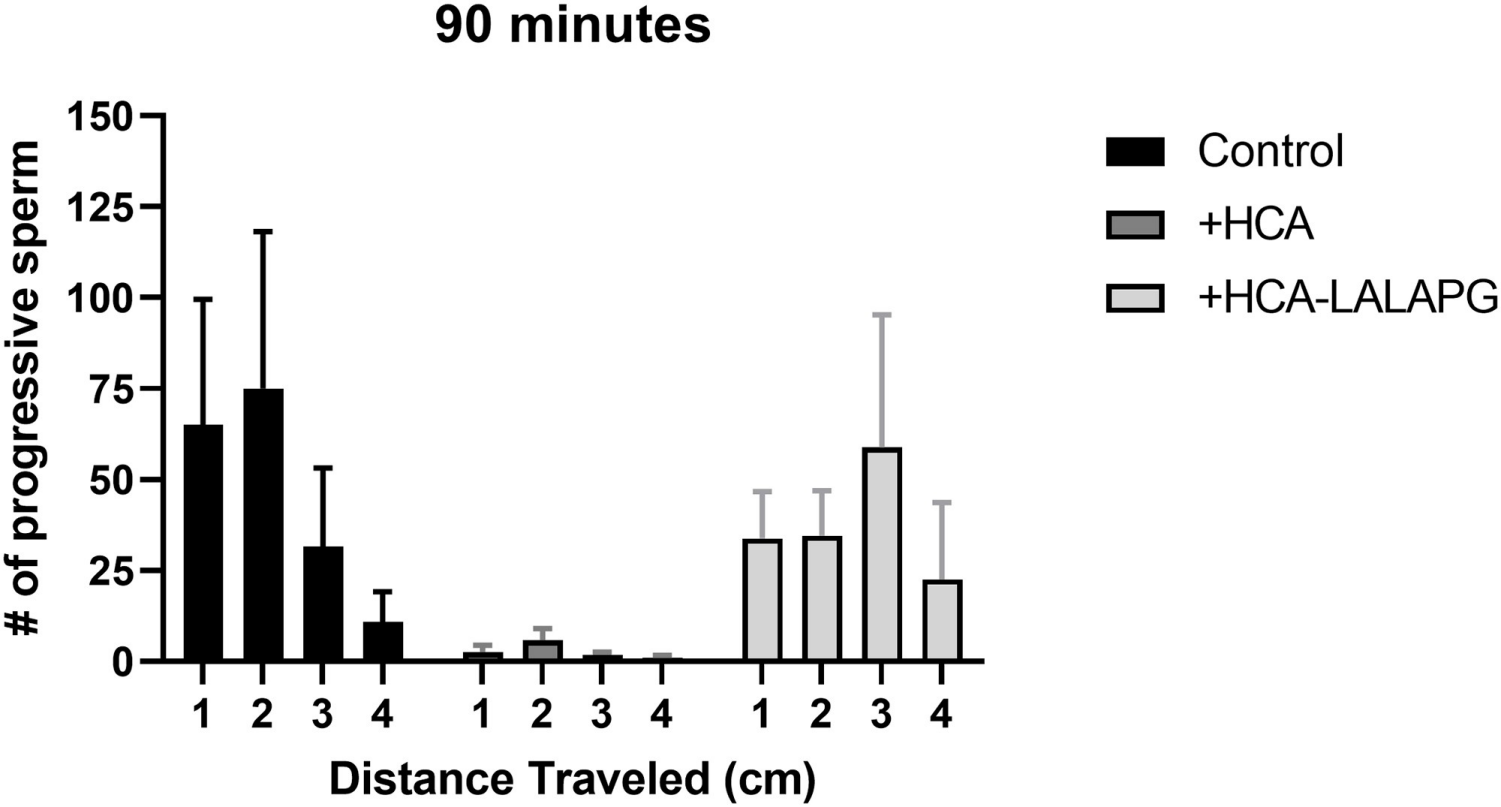
Cervical mucus penetration test with HCA and HCA-LALAPG. The number of progressively motile sperm at 90 minutes in ovulatory cervical mucus was significantly lower in the HCA treatment group than with HCA-LALAPG at all capillary tube depths (1–4 cm). Both antibodies were used at a concentration of 12.5μg/mL. Data are shown as mean ± SEM and is representative of four independent experiments, each with a different cervical mucus donor. Statistical analyses were conducted using a repeated measures ANOVA of log transformed data followed by a Tukey multiple comparisons test. Results were significant when p ≤ 0.05. (* = p ≤ 0.05, ** = p ≤ 0.01, *** = p ≤ 0.001, **** = p ≤ 0.0001).

HCA significantly reduced the penetration of progressive sperm through midcycle cervical mucus at the 30-, 60-, and 90-minute timepoints compared to the no-antibody control and HCA-LALAPG. (S1 Fig and S1 Table). By the 90-minute timepoint, the number of progressively motile sperm in cervical mucus was dramatically lower for HCA-treated sperm than for medium- and HCA-LALAPG-treated sperm at all four penetration depths (Table 1A). The number of progressively motile sperm in cervical mucus after treatment with HCA-LALAPG did not differ significantly from the medium-only control condition and was significantly higher than the number of progressively motile sperm treated with HCA (Table 1B and Fig 4 for specific pairwise comparisons).

**Table 1:** Cervical mucus penetration at 90 minutes with HCA and HCA-LALAPG.

A.				
Condition	90 minutes			
	1cm	2cm	3cm	4cm
Control (no antibody)	65.00±34.59	75.00±43.16	31.5±21.74	10.75±8.43
+HCA	2.5±1.89	5.75±3.33	1.75±0.85	1.00±0.71
+HCA-LALAPG	33.75±12.99	34.5±12.48	58.75±36.54	22.5±21.17
B.				
Comparison	90 minutes			
	1cm	2cm	3cm	4cm
Control vs. HCA	p<0.0001	p = 0.0001	p = 0.002	p = 0.02
Control vs. HCA-LALAPG	p = 0.38	p = 0.75	p = 0.35	p = 0.10
HCA vs. HCA-LALAPG	p<0.0001	p = 0.0007	p<0.0001	p = 0.03

A) Number of progressively motile sperm in the cervical mucus penetration test after 90 minutes (shown as mean ± SEM). B) Statistical differences between treatment groups at each distance after 90 minutes.

The greatest differences between treatments were seen within the first two centimeters (Fig 4). Agglutinated sperm were observed at the entrance of the cervical mucus column (<1 cm depth) with both HCA and HCA-LALAPG treatments, but since the antibody concentration was low, individual sperm were also able to penetrate into the cervical mucus column. In HCA-treated samples, very few progressively motile sperm were observed; the majority of individual sperm appeared to be trapped in the cervical mucus as visualized by rapid vibration of the sperm without any forward motility. HCA-LALAPG also agglutinated sperm at the interface with cervical mucus, but there were far more individual sperm with forward progression. As shown in Table 1A, the number of progressively motile sperm that penetrated the cervical mucus column was comparable between the medium-only control and HCA-LALAPG-treatment groups at 90 minutes; there were significantly fewer progressive sperm in the HCA treatment group compared to both no antibody and HCA-LALAPG (Table 1B). Sperm progression through cervical mucus varied between the four cervical mucus donors (S2 Fig); one subject had inhospitable mucus that did not permit sperm penetration regardless of treatment group, whereas three subjects had hospitable mucus that showed similar patterns of sperm penetration in medium-only control and HCA-LALAPG-treated samples, and significant trapping in HCA-treated samples.

### HCA can induce ADCP whereas HCA-LALAPG cannot

**Fig 5:**
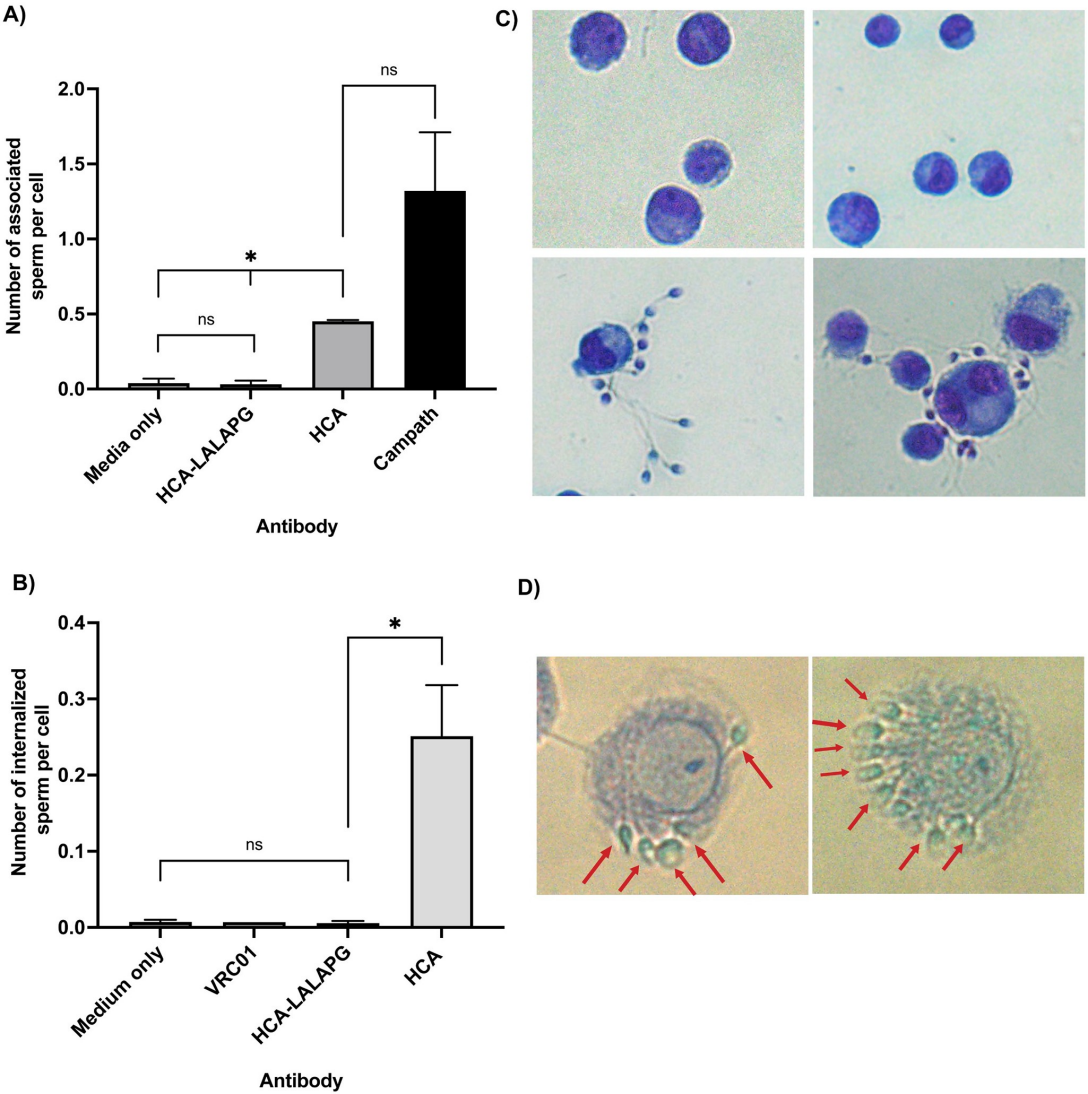
Antibody-dependent sperm phagocytosis. A) Significantly more sperm were associated with U937 macrophages in HCA-treated cultures than in cultures treated with HCA-LALAPG or medium-only control (p = 0.0128, p = 0.0131, respectively). “Associated sperm” were defined as those either attached to the surface of the macrophage (i.e., at the beginning of the phagocytosis process), or inside the cell. Campath, an anti-CD52 monoclonal antibody known to mediate ADCP effector function, was used as a positive control. Final antibody concentrations were 50 μg/mL. B) The number of internalized sperm per macrophage was quantified following a 2.5-hour incubation with antibody-treated sperm. Final antibody concentrations were 50 μg/mL. The number of internalized sperm was significantly higher following treatment with HCA than with HCA-LALAPG, medium-only control, or VRC01 isotype control (p = 0.037; negative control run twice, isotype control run once). (C) Images of macrophages incubated with sperm exposed to either medium-only (top left), HCA-LALAPG (top right), HCA (bottom left), or Campath (bottom right). Images were taken at 200x magnification. (D) Sperm internalization post-HCA treatment (red arrows) by U937 macrophages. Images were taken at 400X magnification. Data are expressed as mean ± SEM of three independently performed experiments. Statistical analyses were conducted using either repeated measures one-way ANOVA or mixed-effects analysis of log-transformed data followed by Tukey multiple comparisons tests.

Fc effector functions of HCA have not previously been fully characterized. We are particularly interested in the capacity of HCA to mediate antibody-dependent cellular phagocytosis (ADCP) since antigen presentation of sperm antigens could lead to antisperm immunity and infertility. Differentiated U937 macrophage-like cells were incubated for 30 minutes with antibody-treated sperm, after which they were washed for 30 minutes in PBS and treated with trypsin for 5 minutes to remove non-specifically bound spermatozoa. Fixed slides were stained with Diff Quik stain kit, a morphology dye stain, and observed under a light microscope to quantify the number of associated spermatozoa per macrophage-like cell. Associated spermatozoa were characterized as those that were either attached to the surface of a cell (i.e., in the beginning of the phagocytosis process), or partially engulfed. The positive control antibody, Campath, elicited the greatest level of sperm association with more than one spermatozoon per effector cell (Fig 5A). The number of sperm associated with U937 macrophages after HCA treatment was ~ 30% of the mean in the Campath condition and was significantly higher than both medium-only control and HCA-LALAPG treatment (p = 0.013 for both comparisons). HCA-LALAPG treatment was not significantly different from the medium-only control (p > 0.99). Attached sperm were clearly visible by microscopy in the HCA- and Campath-treated conditions, while no sperm were visible in the HCA-LALAPG- or medium-only-treated conditions (Fig 5B).

A phagocytosis time-course experiment was conducted to determine whether there was an effect of incubation time on the number of HCA-coated sperm that were phagocytosed by U937 macrophages. Internalized sperm were scored as either partially (head or tail) or wholly phagocytosed. The number of internalized spermatozoa per macrophage increased over time (S3 Fig) and reached a maximum after 2.5 hours (p = 0.018). The number of phagocytosed sperm was significantly higher in HCA treated cultures (p = 0.037) than in medium-only-, HCA-LALAPG-, or VRC01-N- (negative control antibody) treated cultures (Fig 5C). U937 macrophages were occasionally observed with numerous internalized HCA-treated sperm (Fig 5D), whereas this was not observed with HCA-LALAPG, medium or VRC01-N-treatment.

## Discussion

HCA is an antisperm IgG monoclonal antibody currently being studied in preclinical and clinical studies for its contraceptive activity. Here, we characterized an Fc-engineered variant (HCA-LALAPG) to delineate Fab and Fc-mediated functions and to evaluate an alternate vaginal film candidate that may reduce undesirable inflammatory and immune side effects. No differences in sperm agglutination potency were detected. Agglutination, mediated by the Fab region, is thought to be the main contraceptive mechanism of HCA. Both HCA and HCA-LALAPG rapidly agglutinated sperm within 60 seconds [35] even at low antibody concentrations. Thus, engineering and customization of the Fc region of HCA does not affect Fab-mediated sperm agglutination.

Differences in Fc-mediated functions between HCA and HCA-LALAPG were observed. Some antisperm antibodies can immobilize sperm in the presence of a complement source [30, 31]. We have previously shown the ability of HCA to immobilize sperm in a concentration dependent manner in the presence of human serum (complement source) [4]. HCA did not immobilize sperm in the presence of heat-inactivated complement (HiC), demonstrating that sperm immobilization requires enzymatically active complement. With its mutations in the Fc region, HCA-LALAPG has significantly reduced binding to C1q [19] so it was not surprising that it did not immobilize sperm in the presence of complement. While immobilizing sperm may increase HCA’s potential contraceptive ability, complement activation and its cytotoxic effect can also lead to cytokine production and immune cell recruitment [36]. The female reproductive tract is reported to have low levels of complement, so it is unclear how much CDC contributes to the HCA contraceptive effect, but use of HCA-LALAPG could prevent unwanted inflammation in the genital tract, a condition which can increase barrier permeability and pathogen translocation [37]. On the other hand, complement-mediated sperm immobilization could contribute to the contraceptive mechanism of HCA, and the use of HCA-LALAPG could diminish this beneficial effect.

The ability of HCA and the HCA-LALAPG variant to trap sperm in cervical mucus was also compared as this mechanism is thought to contribute to the HCA contraceptive effect. While the exact mechanism of mucus trapping is not fully understood, antibodies may bind to cervical mucus through sugar-sugar hydrogen bonds or Fc receptor-like molecules [38, 39]; other studies indicate that antibodies interact with mucus via weak electrostatic interactions between mucins and the Fc region [10, 11]. As antibody-mucin interactions are weak, transient, and low affinity, it has been difficult to determine the exact mechanism behind mucus trapping. A previous study showed that removing the Fc region on antisperm antibodies decreased cervical mucus trapping [10]. Based on these reports we hypothesized that HCA-LALAPG, with its altered Fc region, would show decreased sperm-cervical mucus interactions. As conception risk is highest around the time of ovulation, midcycle cervical mucus was used for the mucus trapping studies, and a low antibody concentration (12.5μg/mL) was used to increase the likelihood of finding individual motile sperm in the cervical mucus. Nearly all individual (non-agglutinated) HCA-treated sperm were trapped in cervical mucus whereas few HCA-LALAPG-treated sperm were trapped, suggesting that the mutations in the Fc region negatively affect the ability of the HCA-LALAPG variant to interact with cervical mucus. Sperm trapping in cervical mucus could represent an important contraceptive function of HCA, and use of HCA-LALAPG could diminish this function.

Lastly, we demonstrated that HCA, but not HCA-LALAPG, mediates sperm phagocytosis by macrophage-like cells. The ability of HCA to mediate ADCP was previously unknown. The inability of HCA-LALAPG to elicit ADCP was unsurprising since this effector function is dependent upon FcγR binding. HCA-mediated sperm phagocytosis may have serious implications in the context of the female reproductive tract (FRT). Macrophages are widely distributed in the FRT and fluctuate with changes in hormone levels throughout the menstrual cycle [40]. These phagocytic cells make up about 10% of the total number of leukocytes in the FRT [41] and have been shown to be the major mucosal tissue Fc-receptor bearing effector cell and potentially mediate clearance of HIV via interaction with antibodies [42]. As phagocytes are abundant and have potent activity in the FRT, there is the potential for host production of antisperm antibodies following HCA-mediated sperm phagocytosis and antigen presentation. In men with obstructive azoospermia, increased numbers of spermiophages (macrophages that have ingested sperm) have been associated with the formation of autoantibodies against sperm [43]. Host formation of antisperm antibodies would be a highly undesirable immune response in women seeking a reversible method of contraception. However, it is unclear how agglutination contributes to phagocytosis; it is plausible that large agglutinates may be difficult for macrophages to envelop and digest. HCA-LALAPG did not trigger ADCP and is thus less likely to induce immunity to sperm, providing an advantage over HCA in this regard.

Antibody engineering technology continues to improve, offering greater possibilities for antibody design and customization. The challenge is determining the optimal antibody profile in the desired context, especially one as specialized as the vaginal mucosa. Understanding the potential Fc effector functions of an antibody-based drug is critical to the development of a safe and effective product. We have established that the HCA-LALAPG variant had decreased Fc-mediated functions compared to HCA. Some Fc functions such as mucus trapping and sperm immobilization may contribute to the contraceptive efficacy of HCA and may be desirable. Other Fc functions such as ADCP may promote detrimental immune responses through antigen presentation and host production of antisperm antibodies. Fc-engineered variants of HCA may provide safer, more conservative alternatives to HCA, but results from this study indicate that HCA-LALAPG may not be optimal for this role. Use of high resolution maps of FcR binding sites on human IgG1 [22] could provide further insight for designing more specific HCA variants with desired functions. We speculate that the ideal contraceptive antibody candidate would retain an ability to trap sperm in cervical mucus while demonstrating reduced ADCP activity. Functional studies using other Fc variants may be performed in the future as we seek to improve HCA for clinical use.

## Supporting information

S1 Fig Cervical mucus penetration test with HCA and HCA-LALAPG. The number of progressively motile sperm at 30 minutes (A), 60 minutes (B), and 90 minutes (C) in ovulatory cervical mucus, at capillary tube depths of 1, 2, 3 and 4 cm. The number of progressive sperm was significantly lower in the HCA treatment group than with HCA-LALAPG at all time points. Statistics are given in S1 Table. Sperm agglutination was observed for HCA and HCA-LALAPG-treated sperm at the initial centimeter, but non-agglutinated, progressive sperm were commonly observed at 2, 3 and 4 cm with HCA-LALAPG. Data are shown as mean ± SEM and is representative of four independent experiments, each with a different cervical mucus donor. Statistical analyses were conducted using repeated measures ANOVA of log transformed data followed by a Tukey multiple comparisons tests. Results were significant when p ≤ 0.05. (* = p ≤ 0.05, ** = p ≤ 0.01, *** = p ≤ 0.001, **** = p ≤ 0.0001).
(TIF)

S2 Fig Cervical mucus penetration test with HCA and HCA-LALAPG by donor showing the number of progressively motile sperm at 30 minutes, 60 minutes, and 90 minutes in ovulatory cervical mucus. Three out of four donors showed far fewer progressive sperm in the HCA treatment group compared with HCA-LALAPG and medium-only treatment (S2A–S2C Fig) while Donor 104 had fewer progressive sperm in any of the treatment groups (S2D Fig).
(TIF)

S3 Fig Antibody-dependent sperm phagocytosis time-course. The number of internalized sperm per macrophage increased in HCA-treated cultures overtime; a significant increase was observed between 1 hour and 2.5-hour time points (p = 0.018). HCA-LALAPG, medium-only control, and isotype control (VRC01) did not mediate sperm phagocytosis even at the 2.5-hour time point. Final antibody concentrations were 50 μg/mL. Data are expressed as mean ± SEM of three independently performed experiments. Statistical analyses were conducted using repeated measures one-way ANOVA of log-transformed data followed by Tukey multiple comparisons tests.
(TIF)

S1 Table Penetration of ovulatory cervical mucus at 30, 60, and 90 minutes. A) Number of progressively motile sperm in the cervical mucus penetration test (shown as mean ± SEM). B) Statistical differences between treatment groups at each distance. Significant comparisons indicated by shaded cells.
(PDF)

S1 Data (XLSX)
