# Supplementary figures and images for: LALAPG variant of the Human Contraception Antibody (HCA) reduces Fc-mediated effector functions while maintaining sperm agglutination activity
Source: PLoS One. 2023 Mar 30;18(3):e0282147. doi: 10.1371/journal.pone.0282147 (PMC10062632; doi:10.1371/journal.pone.0282147)

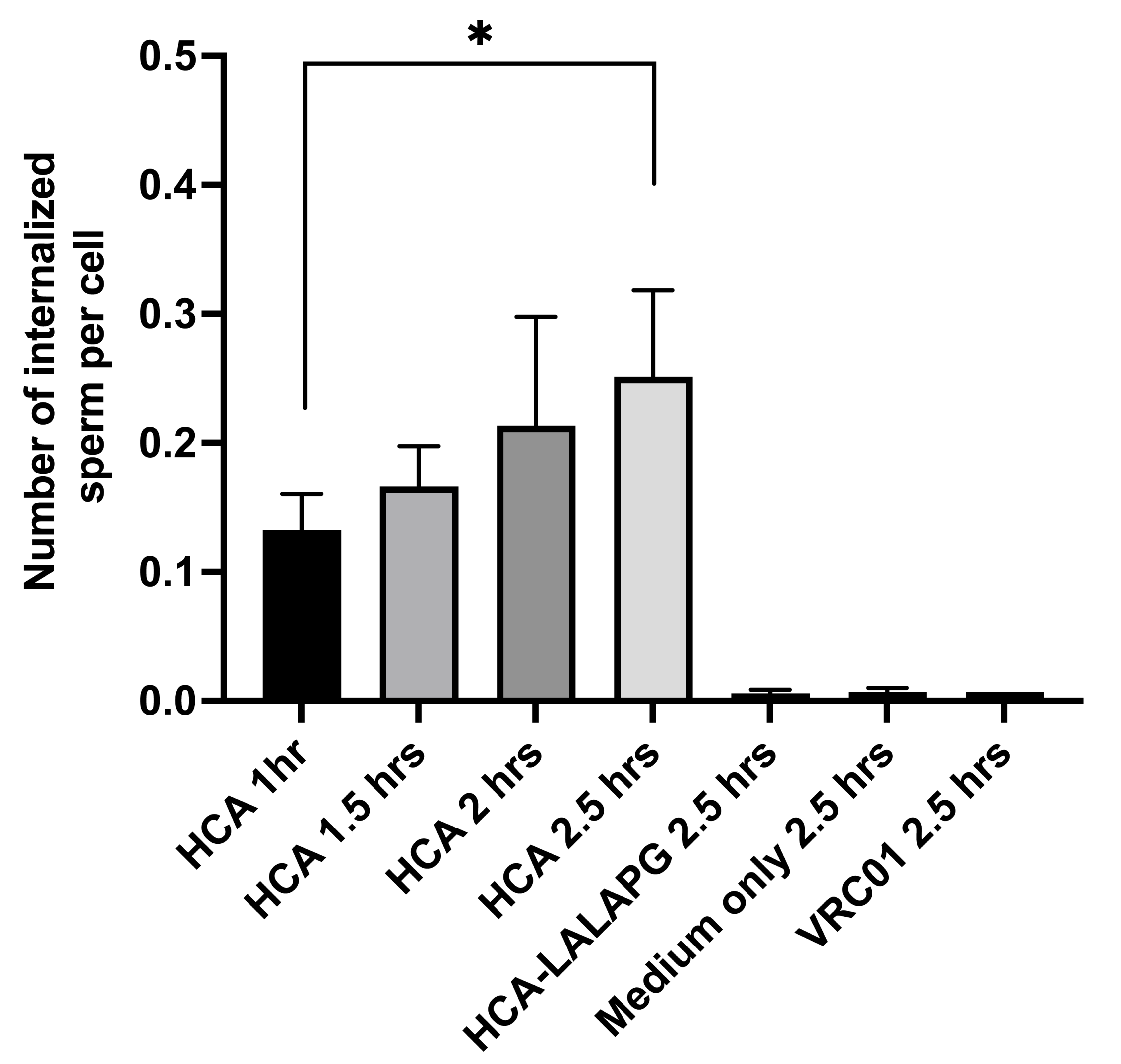

Supplement: S3 Fig — The number of internalized sperm per macrophage increased in HCA-treated cultures overtime; a significant increase was observed between 1 hour and 2.5-hour time points (p = 0.018). HCA-LALAPG, medium-only control, and isotype control (VRC01) did not mediate sperm phagocytosis even at the 2.5-hour time point. Final antibody concentrations were 50 μg/mL. Data are expressed as mean ± SEM of three independently performed experiments. Statistical analyses were conducted using repeated measures one-way ANOVA of log-transformed data followed by Tukey multiple comparisons tests. (TIF) [file pone.0282147.s003.tif]
