# Supplementary material for: LALAPG variant of the Human Contraception Antibody (HCA) reduces Fc-mediated effector functions while maintaining sperm agglutination activity
Source: PLoS One. 2023 Mar 30;18(3):e0282147. doi: 10.1371/journal.pone.0282147 (PMC10062632; doi:10.1371/journal.pone.0282147)
Supplement: S1 Table — A) Number of progressively motile sperm in the cervical mucus penetration test (shown as mean ± SEM). B) Statistical differences between treatment groups at each distance. Significant comparisons indicated by shaded cells. (PDF) [file pone.0282147.s004.pdf]

A)

Condition	30 minutes				60 minutes				90 minutes			
	1cm	2cm	3cm	4cm	1cm	2cm	3cm	4cm	1cm	2cm	3cm	4cm
Control (-HCA)	194.00 ±112.70	37.75 ±13.40	8.00 ±5.05	2.25 ±1.31	84.50 ±27.11	80.25 ±39.93	24.3 ±12.21	3.75 ±2.17	65.00 ±34.59	75.00 ±43.16	31.5 ±21.74	10.75 ±8.43
+HCA	3.25 ±1.97	3.00 ±1.73	4.50 ±4.50	2.75 ±2.43	3.25 ±2.93	2.75 ±2.10	3.25 ±1.70	1.75 ±1.44	2.5 ±1.89	5.75 ±3.33	1.75 ±0.85	1.00 ±0.71
+HCA-LALAPG	17.25 ±8.66	48.25 ±21.09	61.75 ±43.76	24.5 ±14.93	25.5 ±13.32	32.00 ±10.87	68.25 ±33.50	37.00 ±27.88	33.75 ±12.99	34.5 ±12.48	58.75 ±36.54	22.5 ±21.17

B)

Comparison	30 minutes				60 minutes				90 minutes			
	1cm	2cm	3cm	4cm	1cm	2cm	3cm	4cm	1cm	2cm	3cm	4cm
Control vs. HCA	p=0.003	p=0.02	p=0.34	p=0.97	p<0.0001	p<0.0001	p=0.01	p=0.50	p<0.0001	p=0.0001	p=0.002	p=0.02
Control vs. LALAPG	p=0.007	p=0.92	p=0.12	p=0.14	p=0.04	p=0.32	p=0.66	p=0.06	p=0.38	p=0.75	p=0.35	p=0.10
HCA vs. LALAPG	p=0.33	p=0.01	p=0.007	p=0.09	p=0.001	p=0.0004	p=0.002	p=0.006	p<0.0001	p=0.0007	p<0.0001	p=0.03
